# Regional cooperation for mental health in South Asia: Opportunities and challenges

**DOI:** 10.4103/0019-5545.55932

**Published:** 2005

**Authors:** Nimesh G. Desai

The fact that the contemporary science of psychiatry, and indeed mental health, is largely based on western concepts and practice is obvious and well recognized. The process of dissemination of knowledge and expertise from the First World to the Third World, or from the West to the East occurred as part of the larger process of global scientific discourse. The earliest challenge to the practice of the western model of psychiatry arose from South Asia, particularly India, initially on the issue of the practice of psychotherapy,^1^,^2^ and later regarding classification systems^3^ as well as the dynamics of health and illness.^4^ The striking difference in outcome of psychiatric disorders between ‘developed’ and ‘developing’ countries was seen in the first major international collaborative research of the International Pilot Study of Schizophrenia (IPSS).^5^ The collective views from India and other countries in South Asia about the need to recognize the differences had been steadily gaining ground towards the later part of the twentieth century and yet the reference points continued to be Europe and North America, and comparisons were between the West and the East.

The impact of the colonial past had not only been seminal in the development of mental health services and training but also continued in research endeavours. The centrality of the First World remained constant, and comparisons were made from each country with specific countries in North America and Europe. It was, and continues to be, a common experience that practitioners or academicians would be extremely well versed with the details of mental health service delivery systems, training methods or research findings or from a few select countries in the West but know precious little about the same in their own or neighbouring countries. Part of the phenomenon was due to the manner in which various information systems were organized before the Internet came into being. Economic trends and sociocultural patterns also ensured that the interaction among professionals and teams occurred with the central reference point of the First World. If at all the countries in the developing world looked at each other, it was through the eyes of the western world.

The opportunity to learn from one's own neighbours, share experiences to examine similarities and differences, and explore the potential for regional cooperation and collaboration has been realized only very recently. The shared heritage and history, alongside the common social and cultural systems make such efforts easy and, at the same time, the political differences and the colonial mindset render them difficult. As Mike Shooter, President of the Royal College of Psychiatrists concluded while writing the Foreword for a commendable initial effort—*Handbook of psychiatry: A South Asian perspective:* ‘The task for all of us is to help out patients and their carers best as we can, whoever they are. We can only do so properly if we forget our political differences and learn from each other.’^6^

The ability and the willingness to overlook political differences and other mutual biases has been helped by the experience of the South Asian diaspora across the globe, particularly in the countries of the First World. Professionals from neighbouring countries recognized and valued more easily the similarity of mental health perspectives in their personal and professional experiences in the alien world than it would have been possible in their own settings. This realization and the consequent action occurred more readily for psychiatrists and mental health professionals in comparison with their colleagues in other medical specialties, or even other fields of science. The initiative and contribution of some individuals and groups of the South Asian diaspora synchronized with the increasing perception among professionals in the countries of the region that there is much to learn from each other. The benefits of regional cooperation are beginning to be explored through small group interactions as well as large conferences and meetings. The dangers of being focused on the task of organization of large conferences and/or building structures of professional associations are much too real to be ignored. Professional organizations and other such structures at regional levels as well as meetings and conferences have to serve the larger purpose of active cooperation and collaboration. Exchange visits including fellowships, joint exercises in clinical and community settings, training opportunities, multisite research projects, collective scientific publications are some examples of the activities of a meaningful nature, which should be considered.

The very reasons for which the field of psychiatry and mental health have had the advantage of early initiative continue to guide future directions. The possible accusations of individual promotion or ‘scientific tourism’ will merit serious attention. The multiplicity of organizations that have emerged with their focus on regional cooperation is striking. It has to be seriously considered as to what the expected benefits of regional cooperation are, and for whom. The direct benefit of regional cooperation to persons with mental illness and their families through more appropriate mental health service planning,^7^ or the indirect benefit of science and policy through more appropriate research,^8^ have to be the purpose and not positions or trips for individuals! Highly encouraging experiences of regional collaboration in reconstruction of mental health services in the countries of South Eastern Europe belonging to the erstwhile Yugoslavia serve as case study examples of effective regional collaboration,^9^ whereas the accounts of Latin America clearly identify urgent needs of the region.^10^

The South Asian Region has the potential for developing a meaningful and effective model of regional cooperation and collaboration owing to the already existing mental health services, the professional expertise in the region and among the diaspora, the global economic trends in favour of the Asian region, and positive contribution to global mental health, which is based on traditional wisdom, community systems and spiritual depth. The spirit of regional cooperation has sometimes tended to remain elitist, without the large body of professionals being involved in the activities of cooperation, or the benefits reaching the end-users of mental health services.

The globalization sweeping the world in the early part of the twentieth century has helped the process of regional cooperation in many ways. It also poses challenges and opportunities as noted by Okasha: ‘In psychiatry, the most important aspect of globalization is delivering mental health services in an equitable pattern, providing equal treatment and establishing equal outcome to our patient population, no matter which part of the world they come from. To achieve globalization of mental health services, we need more studies on the psychological variables affecting mental illness, the provision of a better structure for psychosocial intervention for both developing and developed countries, and a more equitable distribution of world resources.’^11^

The contribution of regional cooperation and collaboration in global advocacy for appropriate emphasis on mental health is important for the overall health status and for the development of human race.^12^

The old maxim ‘Think globally, and act locally’ needs to be modified to ‘Think globally, collaborate regionally and act locally’ by psychiatrists and mental health professionals all over the world and particularly those in South Asia for the exciting possibilities of contributing to the global mental health challenge and beyond. The opportunities bring commensurate challenges, as they often do, and it is for psychiatrists and mental health professionals in the region, as well as the diaspora, to meet the challenges and fructify the opportunities through collective effort.
